# Serglycin‐induced interleukin‐1β from oesophageal cancer cells upregulate hepatocyte growth factor in fibroblasts to promote tumour angiogenesis and growth

**DOI:** 10.1002/ctm2.1031

**Published:** 2022-08-22

**Authors:** Dongdong Yan, Di Cui, Yun Zhu, Cecilia Ka Wing Chan, Chung Hang Jonathan Choi, Tengfei Liu, Sai Wah Tsao, Stephanie Ma, Annie Lai Man Cheung

**Affiliations:** ^1^ School of Biomedical Sciences Li Ka Shing Faculty of Medicine University of Hong Kong Hong Kong SAR China; ^2^ Center for Clinical Big Data and Analytics The Second Affiliated Hospital School of Medicine Zhejiang University Hangzhou China; ^3^ Department of Biomedical Engineering The Chinese University of Hong Kong Hong Kong SAR China; ^4^ The University of Hong Kong – Shenzhen Hospital Shenzhen China

Dear Editor,

A recent article in this journal highlights the importance of cytokines in the interaction between oesophageal squamous cell carcinoma (ESCC) cells and fibroblasts.^1^ Here, we provide novel insights into the secretion and functional significance of cancer cell‐derived interleukin‐1β (IL‐1β) in the tumour microenvironment (TME) of ESCC.

We previously reported that serglycin (SRGN) regulates midkine (MDK) secretion, and that SRGN‐induced MDK has autocrine stimulatory effects on cancer cells.^2^ Mounting evidence substantiates the important role of SRGN and its glycosaminoglycan (GAG) side chains in regulating secretion of cytokines, enzymes and growth factors,^3^ but its function in the TME of ESCC remains elusive. To study the effects of SRGN‐overexpressing ESCC cells on fibroblasts, we treated human oesophageal fibroblasts (HEFs) with conditioned medium (CM) of ESCC cells that overexpressed wild‐type SRGN, truncated SRGN lacking the GAG attachment domain (ΔGAG), or empty vector (Con).^2^ Western blot and quantitative PCR (q‐PCR) analyses showed that fibroblast activation protein‐α (FAP) was markedly upregulated in SRGN CM‐treated HEFs (Figure 1A). CM from ESCC cells with SRGN‐knockdown (shSRGN CM) produced an opposite effect (Figure 1B). Analysis of RNA‐sequencing data in The Cancer Genome Atlas (TCGA) oesophageal carcinoma dataset revealed positive correlation between SRGN and FAP (Figure S1). Using immunohistochemistry, we further demonstrated that SRGN expression in cancer cells was positively correlated with FAP expression in stromal cells of ESCC (Figure 1C). SRGN CM, but not ΔGAG CM, increased viability (Figure 1D) and migratory ability (Figure 1E) of HEFs. Moreover, HEFs pretreated with SRGN CM facilitated tumour growth and tumour angiogenesis in vivo while ΔGAG CM had no effects (Figure 1F,G).

**FIGURE 1:**
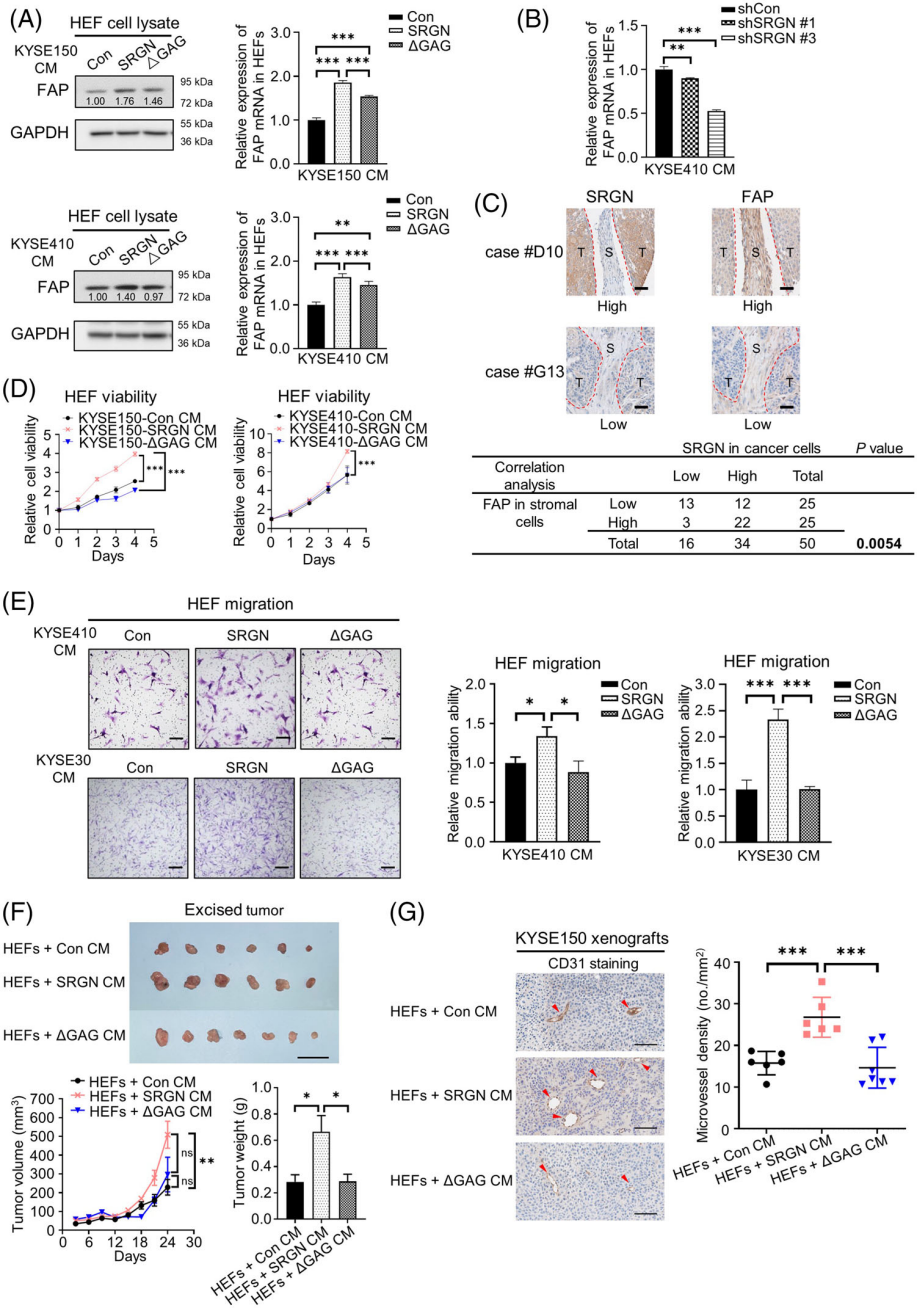
SRGN CM activates HEFs to facilitate tumour growth and angiogenesis in a GAG‐dependent manner. (A) Effects of SRGN CM and ΔGAG CM on FAP expression in HEFs. Left panels, Western blot analysis of FAP expression in HEF cell lysates. Right panels, q‐PCR analysis of the FAP mRNA expression in HEFs (*n* = 3). (B) q‐PCR analysis of the effects of CM from ESCC cells with SRGN‐knockdown on FAP mRNA expression in HEFs (*n* = 3). (C) Immunohistochemical staining of SRGN and FAP in tissue microarray of ESCC (scale bar, 50 μm). T, tumour; S, stroma. The table shows the correlation between SRGN in oesophageal cancer cells and FAP in stromal cells. (D) Effects of SRGN CM and ΔGAG CM on viability of HEFs (*n* = 6). (E) Transwell migration assay was performed to compare the effects of Con CM, SRGN CM, and ΔGAG CM on HEF migration ability (Scale bar, 200 μm; *n* = 4 and 5 for experiments with KYSE410 CM and KYSE30 CM, respectively). (F‐G) Comparison of the effects of HEFs preconditioned by Con CM, SRGN CM or ΔGAG CM on growth and angiogenesis of ESCC tumour xenografts. (F) Upper panel, image of excised xenografts (scale bar, 2 cm). Lower left panel, measurement of tumour volume at different time points. Lower right panel, tumour weight at the end of experiment. *n* = 6 or 7/group (one each from HEFs + Con CM and HEFs + SRGN CM groups was excluded due to necrosis). (G) Representative immunohistochemical images of CD31 expression in sections of tumour xenografts (left panel; scale bar, 100 μm) and the corresponding analysis of microvessel density (right panel; *n* = 6 or 7)

Treatment with recombinant human MDK (rhMDK) activated HEFs (Figure 2A). According to gene ontology (GO) analysis, differentially expressed genes were enriched in biological processes (BP) involving metabolism, ion transport, signalling and motility (Figure 2B). Several most significant GO‐BP terms were consistent with those of colon cancer‐associated fibroblasts (CAFs).^4^ Enriched GO terms in molecular functions and cellular components are presented in Figure S2. KEGG pathway analysis showed that calcium and cAMP signalling pathways, which are related to fibroblast transformation, were enriched (Figure 2C). The second most upregulated gene TMBIM4 (Table S1), which has putative anti‐apoptotic and migration‐promoting functions,^5^, ^6^ might have contributed to increased HEF viability (Figure 2D) and migration (Figure 2E) after rhMDK treatment. Moreover, since MDK‐knockdown abolished the stimulatory effects of SRGN CM on these functions (Figure 2F,G), our data suggest that SRGN‐induced MDK is a novel trigger for HEF activation.

**FIGURE 2:**
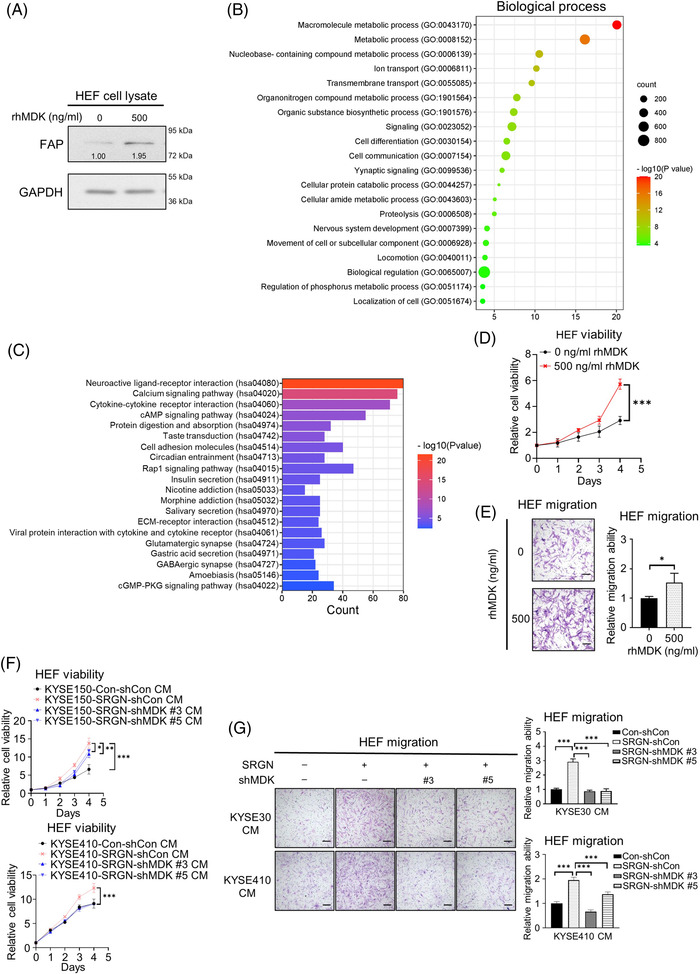
MDK mediates the pro‐viability, pro‐migration and activating abilities of SRGN CM on HEFs. (A) Effect of rhMDK treatment on FAP expression in HEFs. (B) GO analysis of differentially expressed genes (identified by RNA‐sequencing) in HEFs after treatment with rhMDK for 7 days. The top 20 enriched GO terms in the biological process category were shown. (C) Kyoto encyclopaedia of genes and genomes (KEGG) pathway enrichment analysis for differentially expressed genes in rhMDK‐treated HEFs. The top 20 pathways ranked by −log_10_(*P* value) were shown. Effects of rhMDK on (D) viability (*n* = 6) and (E) migration (*n* = 3) of HEFs. Scale bar, 200 μm. Effects of SRGN CM on (F) viability (*n* = 6) and (G) migration (*n* = 3) of HEFs were attenuated by MDK‐knockdown (scale bar, 200 μm)

To determine if SRGN‐overexpressing ESCC cells affect the secretome of HEFs, CM of HEFs treated with SRGN CM was examined using human growth factor arrays (Figure 3A and Figure S3). Western blots showed that amphiregulin (AREG), granulocyte colony‐stimulating factor (G‐CSF), fibroblast growth factor 6 (FGF6) and vascular endothelial growth factor‐D (VEGF‐D) secretions were increased after treatment with either KYSE150‐SRGN CM or KYSE410‐SRGN CM; only hepatocyte growth factor (HGF) was obviously and consistently augmented after treatment with SRGN CM of multiple ESCC cell lines (Figure 3B). A small increase in AREG secretion was detected after treatment with KYSE150‐ΔGAG CM (Figure 3B), but q‐PCR data suggested that SRGN CM increased HGF and AREG transcription in a GAG‐dependent manner (Figure 3C). HGF and AREG mRNAs were reduced upon SRGN‐knockdown (Figure 3D). Since rhMDK treatment elicited only a slight increase in HGF mRNA expression in HEFs and had no effect on AREG (Figure 3E), human cytokine arrays were used to identify the inducers of HGF and AREG (Figure 3F). Among the top three upregulated cytokines in SRGN CM (Figure S4 and Figure 3G), further analyses suggest that IL‐1β acts as a paracrine mediator in SRGN CM to induce transcription of HGF and AREG in HEFs (Figure 3H,I). IL‐18 had little effect (Figure S5). Co‐immunoprecipitation assay showed that intracellular IL‐1β was precipitated with wild‐type SRGN but not with ΔGAG (Figure 3J). Positive correlations were found between the mRNAs of SRGN and IL‐1β, SRGN and HGF, and between IL‐1β and AREG (Figure S1).

**FIGURE 3:**
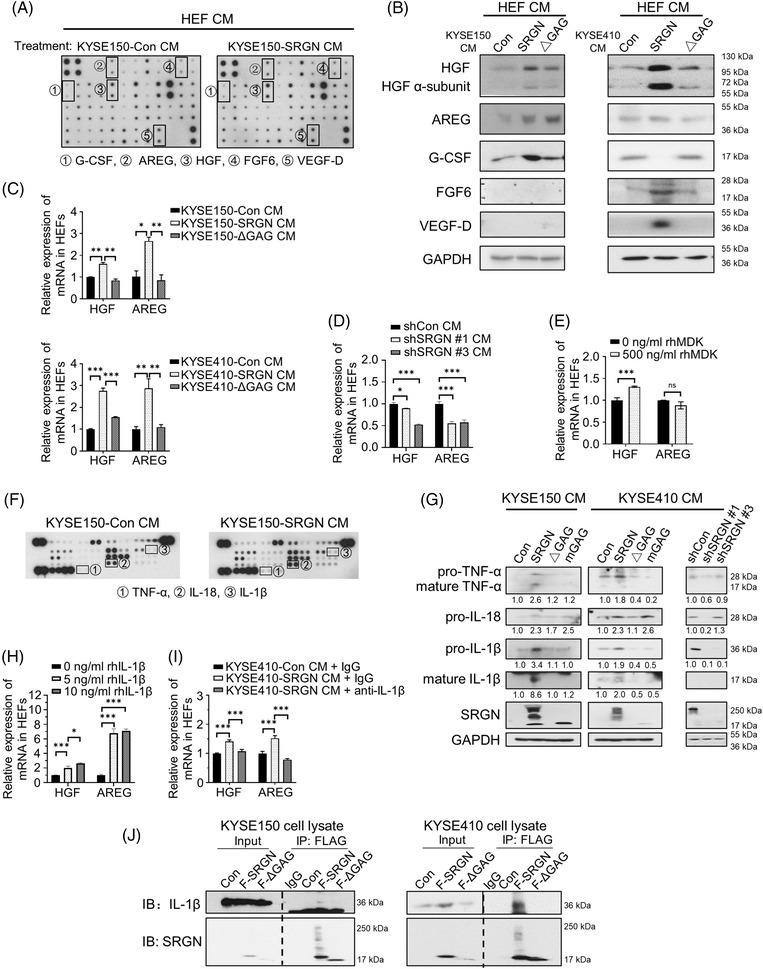
SRGN‐induced IL‐1β upregulates HGF and AREG in HEFs. (A) Growth factor profiling of CM of HEFs pretreated with KYSE150‐Con CM and KYSE150‐SRGN CM for 7 days. (B) Western blot validation of upregulated growth factors indicated by the black frames in the arrays. (C) The mRNA expression levels of HGF and AREG in HEFs treated with indicated CM were examined by q‐PCR (*n* = 3). (D) Q‐PCR examination of the effects of shSRGN CM on HGF and AREG mRNA expression in HEFs (*n* = 3). (E) Effects of rhMDK treatment for 24 h on HGF and AREG mRNA expression in HEFs (*n* = 3). (F) Cytokine array analysis of KYE150‐Con CM and KYSE150‐SRGN CM. The top three upregulated cytokines were indicated with numbers. (G) TNF‐α, IL‐18 and IL‐1β in Con CM, SRGN CM, ΔGAG CM, mGAG CM, shCon CM, and shSRGN CM were examined by Western blot. (H) Dose‐dependent effects of rhIL‐1β treatment (24 h) on HGF and AREG mRNA expression in HEFs (*n* = 3). (I) Effects of antibody neutralization of IL‐1β in SRGN CM on HGF and AREG mRNA expression in HEFs (*n* = 3). (J) Co‐IP of SRGN and IL‐1β using ESCC cell lysates

The CM of HEFs pretreated with SRGN CM promoted angiogenesis in vitro, but the effect was abolished by immunoneutralization of HGF in the HEF CM (Figure 4A,B). Interestingly, Figure 1G suggests that a GAG‐binding molecule was involved in mediating tumour angiogenesis since the tumours in the ΔGAG CM group did not show increased microvessel density. It is well established that activation of caspase 1 by inflammasome activation induces secretion of IL‐1β in mature form,^7^ but the constitutive secretion of its precursor in the absence of inflammasome activation is still unknown. Figure 3G shows that the secreted levels of pro‐IL‐1β and mature IL‐1β were higher in the SRGN CM than in the Con CM, ΔGAG CM or mGAG (SRGN with mutated GAG attachment domain) CM. Since IL‐1β directly interacts with glycosylated SRGN (Figure 3J), as in the case of MDK,^2^ SRGN‐induced IL‐1β may be transported out of the cancer cells to the TME via interaction with the GAG side chains of SRGN. After secretion, pro‐IL‐1β in extracellular space may be processed into active form by matrix metalloproteinases,^8^ the expression and secretion of which can be induced by SRGN.^2^

**FIGURE 4:**
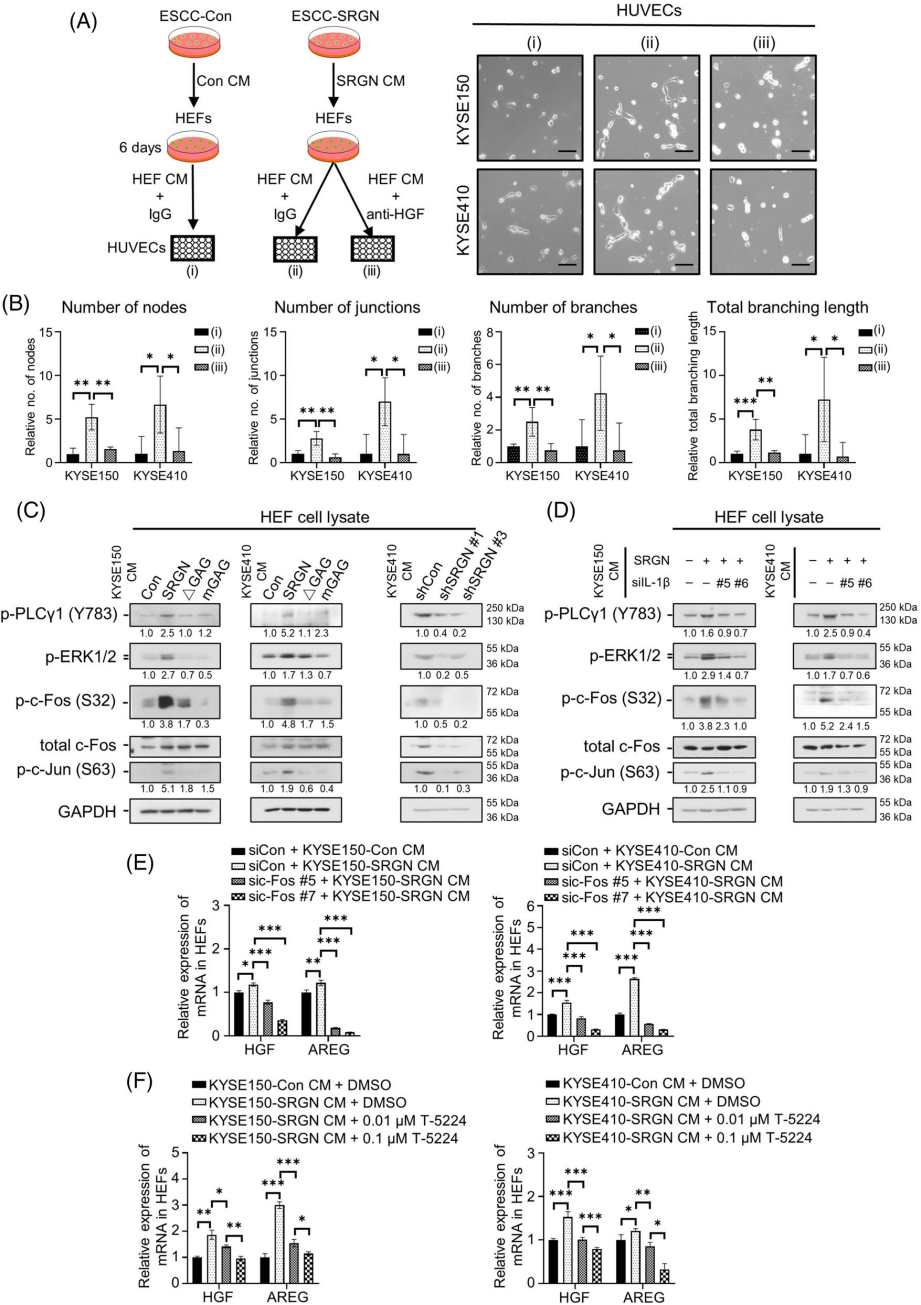
SRGN‐induced IL‐1β upregulates pro‐angiogenic HGF and AREG in HEFs by activating PLCγ1/ERK/AP‐1 pathway. (A) Experimental design (left panel) and representative images of the tube formation assay (right panel; scale bar, 100 μm). (B) Analysis of the tube formation ability of human umbilical vein endothelial cells (HUVECs) using parameters of number of nodes, junctions, branches, and total branching length (*n* = 4). (C) Effects of SRGN CM, ΔGAG CM, mGAG CM, and shSRGN CM on PLCγ1/ERK/AP‐1 signalling pathway in HEFs. (D) Effects of IL‐1β‐knockdown on SRGN CM‐mediated activation of PLCγ1/ERK/AP‐1 signalling pathway in HEFs. Effects of (E) c‐Fos‐knockdown (*n* = 3) and (F) T‐5224 treatment (*n* = 3) on mRNA expression levels of HGF and AREG in HEFs treated with SRGN CM from ESCC cells

Activator protein 1(AP‐1), composed of c‐Fos and c‐Jun, is a transcription factor of HGF and AREG.^9^, ^10^ To determine if AP‐1 was involved in HGF and AREG upregulation in HEFs, HEFs were treated with various CM. Western blotting showed that shSRGN CM suppressed phosphorylation of phospholipase C gamma 1(PLCγ1), ERK, c‐Fos and c‐Jun, while SRGN CM had the opposite effect (Figure 4C). Notably, the effect of SRGN CM on PLCγ1/ERK/AP‐1 pathway was attenuated after IL‐1β‐knockdown (Figure 4D). c‐Fos‐knockdown or treatment with a c‐Fos inhibitor, T‐5224, offset the stimulatory effect of SRGN CM on HGF and AREG mRNA expression (Figure 4E,F), and on HEF activation (Figure S6).

In conclusion, IL‐1β and MDK secreted from SRGN‐overexpressing ESCC cells instigate fibroblasts to acquire CAF phenotypes and create a tumour‐supporting milieu (Figure S7). The involved mediators and their molecular pathways may represent promising targets for oesophageal cancer therapy.

## CONFLICT OF INTEREST

The authors declare no conflict of interest.

## Supporting information



Supporting Information

Supporting Information

Supporting Information

Supporting Information

Supporting Information

Supporting Information

Supporting Information

Supporting Information

Supporting Information

Supporting Information
