# Corrigendum to “Serglycin‐induced interleukin‐1β from oesophageal cancer cells upregulate hepatocyte growth factor in fibroblasts topromote tumour angiogenesis and growth”

**DOI:** 10.1002/ctm2.1185

**Published:** 2023-01-23

Yan D, Cui D, Zhu Y, et al. Serglycin‐induced interleukin‐1βfromoesophageal cancer cells upregulate hepatocyte growth factor in fibroblasts to promote tumour angiogenesis and growth. *Clin Transl Med*.2022;12:e1031. https://doi.org/10.1002/ctm2.1031

Funding information has been included in the acknowledgement section.

The Funding Information details has been replaced in the acknowledgement section as follows:

“This project was supported by the General Research Fund from the Research Grants Council of the Hong Kong SAR, China (Project No. 17100819). We acknowledge Prof. A. D. Theocharis (University of Patras, Greece) for serglycin antibody, Prof. Yutaka Shimada (University of Toyama, Toyama, Japan), and DSMZ for the KYSE cell lines. We thank Centre for PanorOmic Sciences of Li Ka Shing Faculty of Medicine, University of Hong Kong for the support in RNA‐sequencing and in analysis of tissue microarray.”

Author apologizes for this error

