# Supplementary material for: Serglycin‐induced interleukin‐1β from oesophageal cancer cells upregulate hepatocyte growth factor in fibroblasts to promote tumour angiogenesis and growth
Source: Clin Transl Med. 2022 Aug 22;12(8):e1031. doi: 10.1002/ctm2.1031 (PMC9394751; doi:10.1002/ctm2.1031)
Supplement: Supplementary file 1 — Supporting Information [file CTM2-12-e1031-s001.docx]

**Supporting Information**

**Supplementary Materials and methods**

**Recombinant proteins and small molecule inhibitor**

Recombinant human midkine (rhMDK, #450-16, PeproTech, Rehovot, Israel), interleukin-18 (rhIL-18, #9124-IL, R&D Systems, Minneapolis, MN, USA) and interleukin-1β (rhIL-1β, #201-LB/CF, R&D Systems) were reconstituted according to manufacturers’ guidelines. A small molecule inhibitor T-5224 (#S8966, SelleckChem, Houston, TX, USA) was used in this study to inhibit activating protein-1 (AP-1).

**Cell lines**

Human ESCC cell lines, KYSE30, KYSE150 and KYSE410 (DSMZ, Braunschweig, Germany) were used in this study. KYSE150 was derived from a poorly differentiated ESCC resected from a patient after radiotherapy, while KYSE30 and KYSE410 were both derived from poorly differentiated invasive ESCC resected from patients prior to treatment ^1^. The KYSE cell lines were maintained in RPMI1640 (#R6504, Sigma-Aldrich, St Louis, MO, USA) supplemented with 10% fetal bovine serum (FBS, #10270106, Gibco, Thermo Fisher Scientific, Waltham, MA, USA). All ESCC cell lines were authenticated using short tandem repeat analysis and checked routinely for mycoplasma. HEFs (#2730, ScienCell Research Laboratories, Carlsbad, CA, USA) were cultured in fibroblast medium (FM, #2301-b, ScienCell Research Laboratories) supplemented with 1% fibroblast growth supplement (FGS, #2352, ScienCell Research Laboratories) and 2% FBS. Human umbilical vein endothelial cells (HUVECs, #C-003-5C, Thermo Fisher Scientific) were cultured in Medium 200PRF (#M-200PRF-500, Thermo Fisher Scientific) supplemented with 2% low serum growth supplement (LSGS, #S-003-10, Thermo Fisher Scientific). Cell cultures were incubated at 37°C in 5% CO_2._

**Gene overexpression and silencing**

Stable expression cell lines were constructed through lentiviral infection to overexpress empty vector (Con), wild-type SRGN (SRGN), truncated SRGN without a part of C-terminus containing the domain for GAG attachment (ΔGAG), SRGN with all eight serine residues in the GAG attachment domain mutated to alanine (mGAG), FLAG-fusion SRGN (F-SRGN) and FLAG-fusion ΔGAG (F-ΔGAG) as previously described ^2^. Gene knockdown was achieved by using shRNAs or siRNAs, with empty vector pLKO.1 (shCon) or scrambled siRNA sequences (siCon) as controls respectively. The clones with shRNA sequences against SRGN and MDK (**Table S2**) were obtained from Sigma-Aldrich, and used to establish stable knockdown cell lines. For transient transfection, two siRNAs targeting IL-1β (siIL-1β #5, SI03059966; siIL-1β #6, SI03095701) and two targeting c-Fos (sic-Fos #5, SI02781429; sic-Fos #7, SI03066028), obtained from Qiagen (Hilden, Düsseldorf, Germany), were transfected using Lipofectamine^TM^ RNAiMAX Transfection Reagent (#13778150, Thermo Fisher Scientific) following the manufacturer’s manual. Validations of successful knockdowns are shown in **Figure S8**.

**Collection of conditioned media**

When the cells reached about 50% confluence, culture medium was replaced by serum-free medium. Forty-eight hours later, the conditioned medium (CM) was collected, filtered with 0.45 μm filter to remove the cells, and stored at -80 °C for further use. For Western blot, the CM was concentrated at least 20-fold using Amicon^®^ Ultra – 4 mL Centrifugal Filters Ultracel^®^ - 3K (#UFC800396, Millipore, Billerica, MA, USA). For experiments that utilized fibroblasts pretreated with the CM from ESCC cells, the CM was diluted with an equal volume of culture medium supplemented with 1% FBS for cell viability assays, or with FBS-free culture medium for signaling pathway analysis. For immunoneutralization of HGF and IL-1β, the CM was incubated with respective antibodies (mouse anti-HGF, #MAB294, R&D Systems or mouse anti-IL-1β, #MAB201, R&D Systems) for 24 hours before use.

**Cytokine and growth factor arrays**

The undiluted conditioned media of KYSE150-Con and KYSE150-SRGN cells were used to compare secretion of 36 different cytokines using Proteome Profiler Human Cytokine Array Kit (#ARY005B, R&D Systems) according to the manufacturer’s protocol. Human Growth Factor Antibody Array C1 (#AAH-GF-1-4, RayBiotech, Peachtree Corners, GA, USA) which detects 41 different growth factors was used to compare the CM of HEFs exposed to CM of KYSE150-Con cells for 14 days with that of HEFs treated with CM of KYSE150-SRGN cells.

**Western blot and co-immunoprecipitation**

For Western blot, cell lysates were prepared using RIPA Lysis and Extraction Buffer (#89900, Thermo Fisher Scientific) supplemented with cOmplete™, Mini, ethylenediaminetetraacetic acid-free Protease Inhibitor Cocktail (#04693159001, Roche, Basel, Switzerland) and PhosSTOP™ phosphatase inhibitor cocktail (#04906837001, Roche). The details of protein extraction were described previously ^3^. The protein concentrations were measured using Pierce™ BCA Protein Assay Kit (#23227, Thermo Fisher Scientific). For co-immunoprecipitation (co-IP), cell lysates were prepared using IP lysis buffer (50 mM Tris, 150 mM NaCl, 0.5% NP-40, 5 mM ethylenediaminetetraacetic acid) supplemented with protease and phosphatase inhibitors as described above. The co-IP assays were performed using anti-FLAG M2 affinity gel beads (#A2220-5ML, Sigma-Aldrich) according to manufacturer’s instruction. Details of immunoblotting were described previously ^3^. Details of the antibodies used in Western blot are given in **Table S3**. The signals were detected using Fuji medical x-ray film (#4741023951, Fujifilm, Tokyo, Japan) or Amersham Hyperfilm ECL (#28-9068-39, Chicago, IL, USA), and Clarity Western ECL Substrate (#1705061, Bio-Rad Laboratories) or SuperSignal™ West Femto Maximum Sensitivity Substrate (#34095, Thermo Fisher Scientific). Intensities of protein bands on developed films were quantified by ImageJ and normalized to that of glyceraldehyde 3-phosphate dehydrogenase (GAPDH).

**RNA extraction, complementary DNA synthesis and quantitative real-time polymerase chain reaction**

Total RNA was extracted using TRIzol (#15596018, Thermo Fisher Scientific) or RNeasy Mini Kit (#74104, Qiagen) following the manufacturers’ protocols. Complementary DNA was obtained by using High-Capacity cDNA Reverse Transcription Kit (#4374966, Thermo Fisher Scientific). Quantitative real-time polymerase chain reaction (q-PCR) was conducted by using iTaq universal SYBR green supermix (#1725124, Bio-Rad Laboratories, Hercules, CA, USA). Relative gene expression values were calculated as 2^−ΔΔCt^ which represents the fold change compared with GAPDH. Primers used are listed in **Table S4**.

**RNA sequencing**

RNA sequencing was carried out at the Centre for PanorOmic Sciences of the University of Hong Kong. Ribosomal RNA depleted RNA library was prepared using NEBNext^®^ rRNA Depletion Kit (Human/Mouse/Rat) (#E6310, New England Biolabs, Ipswich, MA, USA) and NEBNext^®^ Ultra^TM^ II Directional RNA Library Prep Kit for Illumina^®^ (#E7760, New England Biolabs). Paired-end sequencing was conducted on an Illumina NovaSeq 6000 system (San Diego, CA, USA). Each sample generated an average throughput of 9.4 GB (62 M reads). Around 94% of the bases reached Q30, which denotes the accuracy of a base call to be 99.9%. Alignment and expression analysis were performed by using RSEM 1.2.31 and EBSeq 1.18.0. The results were presented as transcripts per million. With a fold change of 1.5 as cut-off, differentially expressed genes were subjected to Gene Ontology (GO) and Kyoto encyclopedia of genes and genomes (KEGG) analyses using PANTHER GO-slims (<http://geneontology.org/>) ^6^ and the database for annotation, visualization and integrated discovery (DAVID) (https://david.ncifcrf.gov/summary.jsp) ^4,5^ respectively.

**Cell viability assay**

Resazurin reduction assay was conducted to measure cell viability. In brief, cells were incubated in culture medium containing 0.02% (w/v) resazurin sodium salt (#R7017, Sigma-Aldrich) for 4 hours at 37°C. The fluorescence (570 nm excitation and 600 nm emission) was read on a multilabel plate reader (Varioskan^®^ Flash, #5250040, Thermo Fisher Scientific).

**Cell migration assay**

Transwell migration assay was performed as previously described ^7^. Briefly, 5 × 10^4^ HEFs resuspended in serum- and FGS-free FM were seeded into the upper chamber (#353097, Corning, Corning, NY, USA). Conditioned medium (collected from ESCC cell lines originally derived from invasive ESCC, i.e. KYSE30 and KYSE410) or rhMDK was added to the lower chamber (#353504, Corning). Six hours later, the cells in the upper chambers were wiped off with cotton swabs and cells that migrated to lower surface of the membrane of the upper chamber were stained with crystal violet. Five fields of each membrane under 10× objective lens were captured and the area of stained cells was quantified using ImageJ software.

**Endothelial tube formation assay**

Prior to the endothelial tube formation assay, HUVECs were cultured in Medium 200PRF supplemented with 0.4% LSGS for 24 hours. The HUVECs were then suspended in different conditioned media as indicated and seeded in a 24-well plate which was pre-coated with Geltrex™ Reduced Growth Factor Basement Membrane Matrix (#12760-021, Thermo Fisher Scientific). After incubation at 37°C in 5% CO_2_ for 6 hours, capillary-like structures in six random fields from each well were imaged and the quantification of tube formation ability was conducted using ImageJ software.

**Tumor xenograft experiment**

About 2 × 10^5^ HEFs that were pretreated with CM of KYSE150-Con, KYSE150-SRGN or KYSE150-ΔGAG cells for 7 days were mixed with 5 × 10^5^ KYSE150 cells in equal volumes of PBS and Matrigel^®^ Basement Membrane Matrix (#354234, Corning). One hundred μl of the mixture were injected subcutaneously into the right flank of 6-week-old BALB/c female nude mice (n = 7/group) to establish tumor xenografts. The tumor volume (volume = 0.5 × length × width^2^) ^2^ was measured every three days. At the end of the experiment (i.e. 24 days after injection), the tumors were excised and the tumor wet weight was recorded. After fixation in 4% paraformaldehyde and embedding in paraffin, 5 μm sections of the tumors were cut and processed for immunohistochemistry. The protocol for the xenograft experiment was approved by the Committee on the Use of Live Animals in Teaching and Research of the University of Hong Kong.

**Immunohistochemistry**

Immunohistochemistry was performed as previously described ^2^. A tissue microarray (TMA) containing 50 cases of human esophagus squamous cell carcinoma tissue (#HEso-Squ127Lym-01, US Biomax, Derwood, MD, USA) were immunostained for SRGN and FAP. Rabbit anti-SRGN ^8^, a gift from Professor Achilleas D Theocharis (University of Patras, Patras, Greece), and other antibodies used for immunohistochemistry are listed in **Table S5**. The expression level of SRGN in the cancer cells and FAP in the stroma were represented by histoscore scores on an arbitrary scale: 0, no immunoreactivity; 1, weak; 2, moderate and 3, strong. The scores were then grouped into low (scores 0-1) and high (scores 2-3) categories. Microvessel density (MVD) was calculated based on immunohistochemical staining of CD31 as described previously ^9^. First, areas with the largest numbers of CD31-positive microvessels were selected in sections scanned at low magnification (40 ×). Then MVD was counted as the average number of microvessels from ten different fields at 200 × magnification in the selected area. A CD31-positive endothelial cell or cluster separable from adjacent vessels was regarded as one countable microvessel.

**Analysis of gene expression using cancer patient dataset**

The RNA-sequencing data of esophageal carcinoma (ESCA) cohort were obtained from The Cancer Genome Atlas (TCGA) database in Genomic Data Commons Data Portal, National Cancer Institute using University of California Santa Cruz’s Xena browser (https://xenabrowser.net/). These data were analyzed for correlations between gene expression levels.

**Statistical analysis**

All *in vitro* experiments were repeated at least three times and the results shown as the mean ± standard deviation. The data were analyzed by Student’s *t*-test using IBM SPSS Statistics 25 (IBM, Chicago, IL, USA). Pearson’s correlation analysis was used to determine the correlations between gene expression levels. The correlation between the protein levels in TMA was analyzed by Fisher’s Exact test. Differences were considered significant when *P* values were less than 0.05 (*, *P* < 0.05; **, *P* < 0.01; ***, *P* < 0.001).

**References**

1. Shimada Y, Imamura M, Wagata T, Yamaguchi N, Tobe T. Characterization of 21 newly established esophageal cancer cell lines. *Cancer*. 1992;69(2):277-284.

2. Zhu Y, Lam AK, Shum DK, et al. Significance of serglycin and its binding partners in autocrine promotion of metastasis in esophageal cancer. *Theranostics*. 2021;11(6):2722.

3. Hui CM, Cheung PY, Ling MT, et al. Id‐1 promotes proliferation of p53‐deficient esophageal cancer cells. *Int J Cancer*. 2006;119(3):508-514.

4. Dennis G, Sherman BT, Hosack DA, et al. DAVID: database for annotation, visualization, and integrated discovery. *Genome Biol*. 2003;4(9):1-11.

5. Da Wei Huang BTS, Stephens R, Baseler MW, Lane HC, Lempicki RA. DAVID gene ID conversion tool. *Bioinformation*. 2008;2(10):428.

6. Mi H, Muruganujan A, Ebert D, Huang X, Thomas PD. PANTHER version 14: more genomes, a new PANTHER GO-slim and improvements in enrichment analysis tools. *Nucleic Acids Res*. 2019;47(D1):D419-D426.

7. Li B, Tsao SW, Li YY, et al. Id‐1 promotes tumorigenicity and metastasis of human esophageal cancer cells through activation of PI3K/AKT signaling pathway. *Int J Cancer*. 2009;125(11):2576-2585.

8. Theocharis AD, Seidel C, Borset M, et al. Serglycin constitutively secreted by myeloma plasma cells is a potent inhibitor of bone mineralization in vitro. *J Biol Chem*. 2006;281(46):35116-35128.

9. Du Y, Zhang J-y, Gong L-p, et al. Hypoxia-induced ebv-circLMP2A promotes angiogenesis in EBV-associated gastric carcinoma through the KHSRP/VHL/HIF1α/VEGFA pathway. *Cancer Lett*. 2021;
