# Supplementary material for: Serglycin‐induced interleukin‐1β from oesophageal cancer cells upregulate hepatocyte growth factor in fibroblasts to promote tumour angiogenesis and growth
Source: Clin Transl Med. 2022 Aug 22;12(8):e1031. doi: 10.1002/ctm2.1031 (PMC9394751; doi:10.1002/ctm2.1031)
Supplement: Supplementary file 2 — Supporting Information [file CTM2-12-e1031-s004.docx]

**Supplementary tables**

Table S1. Top 20 of upregulated protein-coding transcripts in rhMDK-treated HEFs

| **Transcript** | **Fold change** |
| --- | --- |
| BLOC1S5-TXNDC5 | 5311.08 |
| TMBIM4 | 3768.63 |
| RGPD2 | 3754.48 |
| SYS1-DBNDD2 | 3353.94 |
| SENP3-EIF4A1 | 2616.59 |
| RP11-432B6.3 | 1999.61 |
| RP11-603J24.9 | 1845.87 |
| RP11-1035H13.3 | 1662.80 |
| RP11-49K24.6 | 1619.31 |
| IRX4 | 1518.17 |
| C8orf44-SGK3 | 1496.93 |
| SST | 1315.88 |
| HIST2H3C | 1201.78 |
| AP003419.11 | 1130.78 |
| APOBR | 1113.59 |
| RP11-108K14.8 | 930.52 |
| NDUFC2-KCTD14 | 881.97 |
| RPL36A-HNRNPH2 | 852.64 |
| AC108938.5 | 840.50 |
| TSNAX-DISC1 | 774.75 |

Table S2. Oligonucleotide sequences of shRNA constructs used in this study

| Clone No. | TRC No. | shRNA sense sequence (5’-3’) |
| --- | --- | --- |
| shMDK #3 | TRCN0000063993 | CCGGCGACTGCAAGTACAAGTTTGACTCGAGTCAAACTTGTACTTGCAGTCGTTTTTG |
| shMDK #5 | TRCN0000303918 | CCGGTGTCTGCTCGTTAGCTTTAATCTCGAGATTAAAGCTAACGAGCAGACATTTTTG |
| shSRGN #1 | TRCN0000007985 | CCGGGCAGAGCTAGTGGATGTGTTTCTCGAGAAACACATCCACTAGCTCTGCTTTTT |
| shSRGN #3 | TRCN0000007987 | CCGGCCAGGACTTGAATCGTATCTTCTCGAGAAGATACGATTCAAGTCCTGGTTTTT |

Table S3. List of antibodies used in Western blot

| Antibodies | Source | Dilution | Company | Catalog number |
| --- | --- | --- | --- | --- |
| **Primary antibodies** |  |  |  |  |
| AREG | Mouse | 1:500 | R&D Systems | MAB262 |
| c-Fos | Rabbit | 1:1000 | Cell Signaling Technology | 4384 |
| c-Jun | Rabbit | 1:1000 | Cell Signaling Technology | 9165 |
| ERK1/2 | Rabbit | 1:1000 | Cell Signaling Technology | 4695 |
| FAP | Rabbit | 1:1000 | Abcam | ab53066 |
| FGF6 | Mouse | 1:500 | Santa Cruz Biotechnology | sc-374518 |
| c-Fos | Rabbit | 1:1000 | Cell Signaling Technology | 4384 |
| GAPDH | Rabbit | 1:50000 | Proteintech Group | 10494-1-AP |
| GCSF | Goat | 1:2000 | R&D Systems | AF-214-NA |
| HGF | Rabbit | 1:200 | Abcam | ab178395 |
| IL-18 | Mouse | 1:1000 | MBL International Corporation | D043-3 |
| IL-1β | Mouse | 1:500 | R&D Systems | MAB201 |
| MDK | Rabbit | 1:5000 | PeproTech | 500-P171 |
| phospho-c-Fos (Ser32) | Rabbit | 1:1000 | Cell Signaling Technology | 5348 |
| phospho-c-Jun (Ser63) | Rabbit | 1:1000 | Cell Signaling Technology | 2361 |
| phospho-ERK1/2 (T202/Y204) | Rabbit | 1:1000 | Cell Signaling Technology | 4370 |
| phospho-PLCγ1 (Y783) | Rabbit | 1:1000 | Cell Signaling Technology | 14008 |
| SRGN | Mouse | 1:500 | Santa Cruz Biotechnology | sc-393521 |
| SRGN | Rabbit | 1:250 | Sigma-Aldrich | HPA000759 |
| TNF-α | Mouse | 1:500 | Santa Cruz Biotechnology | sc-52746 |
| VEGF-D | Mouse | 1:500 | R&D Systems | MAB2861 |
| **Secondary antibodies** |  |  |  |  |
| Anti-mouse IgG, HRP-linked | Horse | 1:2500 | Cell Signaling Technology | 7076 |
| Anti-rabbit IgG, HRP-linked | Goat | 1:2500 | Cell Signaling Technology | 7074 |

Table S4. List of primers used in qPCR

|  | Sequence (5’ – 3’) | |  |
| --- | --- | --- | --- |
| Genes | Forward | Reverse | |
| AREG | GTGGTGCTGTCGCTCTTGATA | CCCCAGAAAATGGTTCACGCT | |
| FAP | TGAACGAGTATGTTTGCAGTGG | GGTCTTTGGACAATCCCATGT | |
| c-Fos | TGGCGTTGTGAAGACCATGA | CTGTCTCCGCTTGGAGTGTA | |
| GAPDH | AAGGTCATCCCTGAGCTGAA | TGACAAAGTGGTCGTTGAGG | |
| HGF | GCTATCGGGGTAAAGACCTACA | CGTAGCGTACCTCTGGATTGC | |
| IL-1β | ATGATGGCTTATTACAGTGGCAA | GTCGGAGATTCGTAGCTGGA | |
| SRGN | CCTCAGTTCAAGGTTATCCTACGC | CGTCTTTGGAAAAAGGTCAGTCCT | |

Table S5. List of antibodies used in immunohistochemistry

| Antibodies | Source | Dilution | Company/Lab | Catalog number |
| --- | --- | --- | --- | --- |
| **Primary antibodies** |  |  |  |  |
| CD31 | Goat | 1:100 | Santa Cruz Biotechnology | sc-1506 |
| FAP | Rabbit | 1:50 | Abcam | ab53066 |
| SRGN | Rabbit | 1.38 μg/ml | Gift from Professor Achilleas D Theocharis |  |
| **Secondary antibodies** |  |  |  |  |
| Anti-goat IgG, HRP-linked | Horse | Ready to use | Vector Laboratories | MP-7405 |
| Anti-mouse IgG, HRP-linked | Goat | Ready to use | Dako | K4000 |
| Anti-rabbit IgG, HRP-linked | Goat | Ready to use | Dako | K4002 |
