# Supplementary figures and images for: Serglycin‐induced interleukin‐1β from oesophageal cancer cells upregulate hepatocyte growth factor in fibroblasts to promote tumour angiogenesis and growth
Source: Clin Transl Med. 2022 Aug 22;12(8):e1031. doi: 10.1002/ctm2.1031 (PMC9394751; doi:10.1002/ctm2.1031)

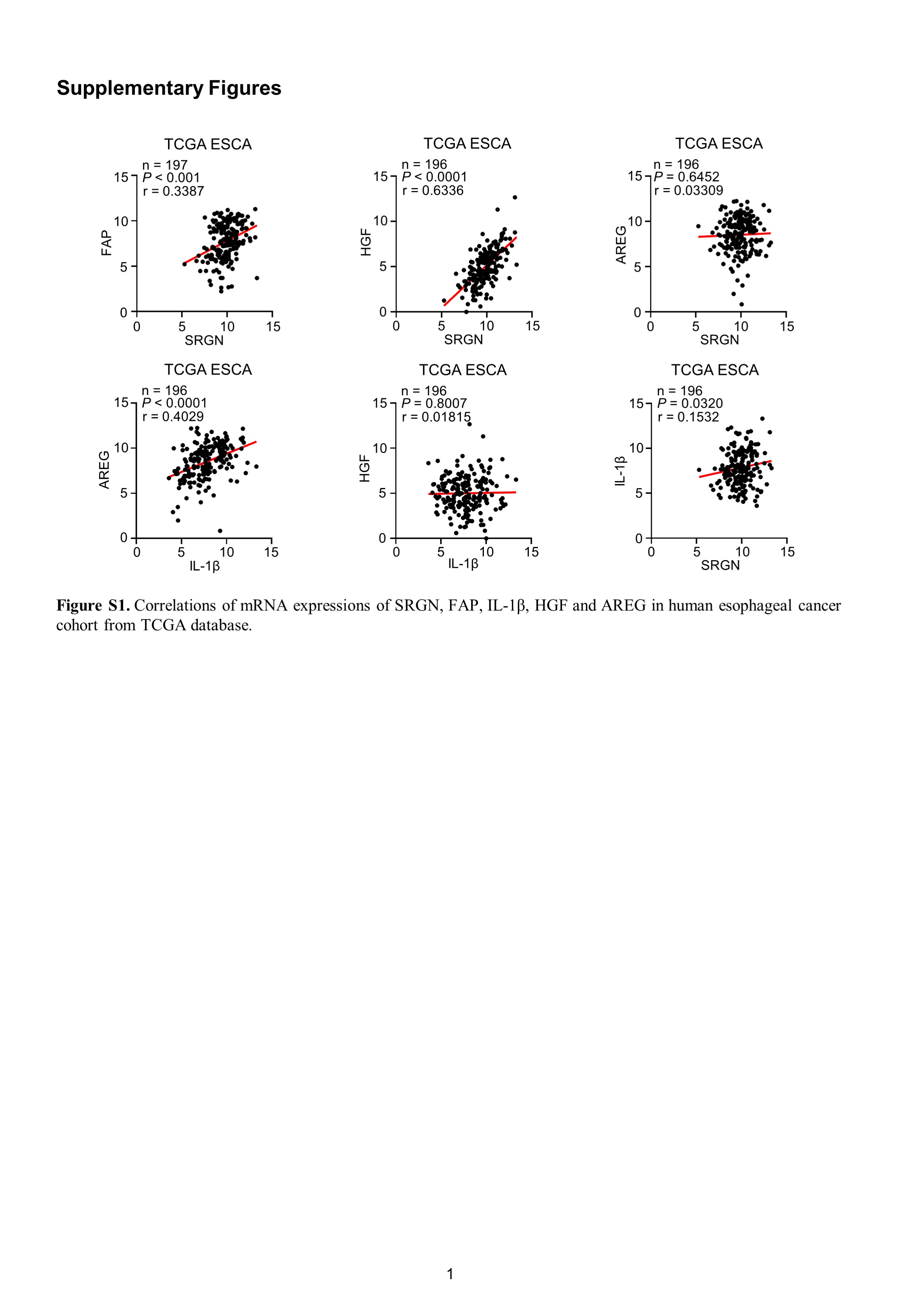

Supplement: Supplementary file 3 — Supporting Information [file CTM2-12-e1031-s002.jpg]

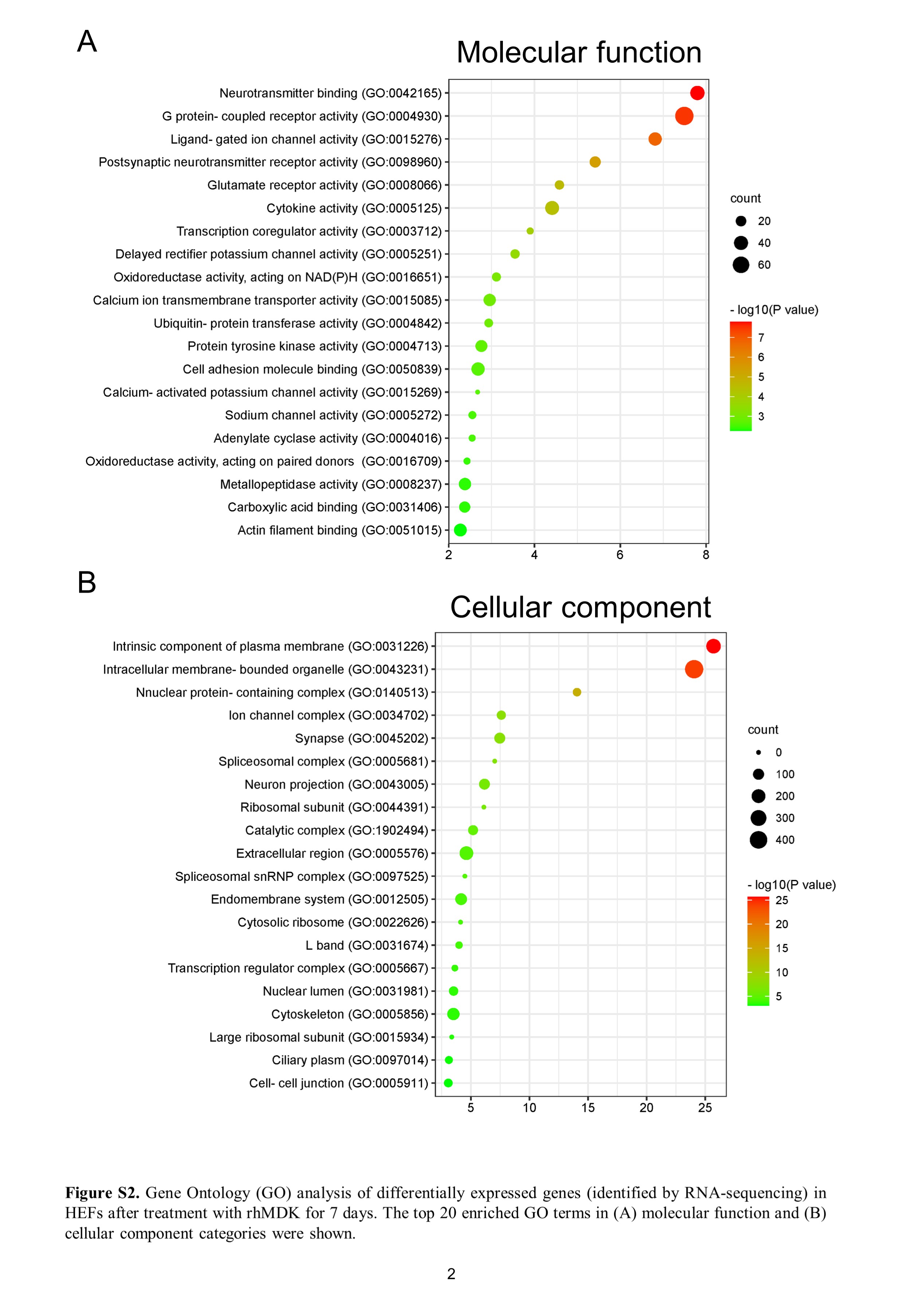

Supplement: Supplementary file 4 — Supporting Information [file CTM2-12-e1031-s003.jpg]

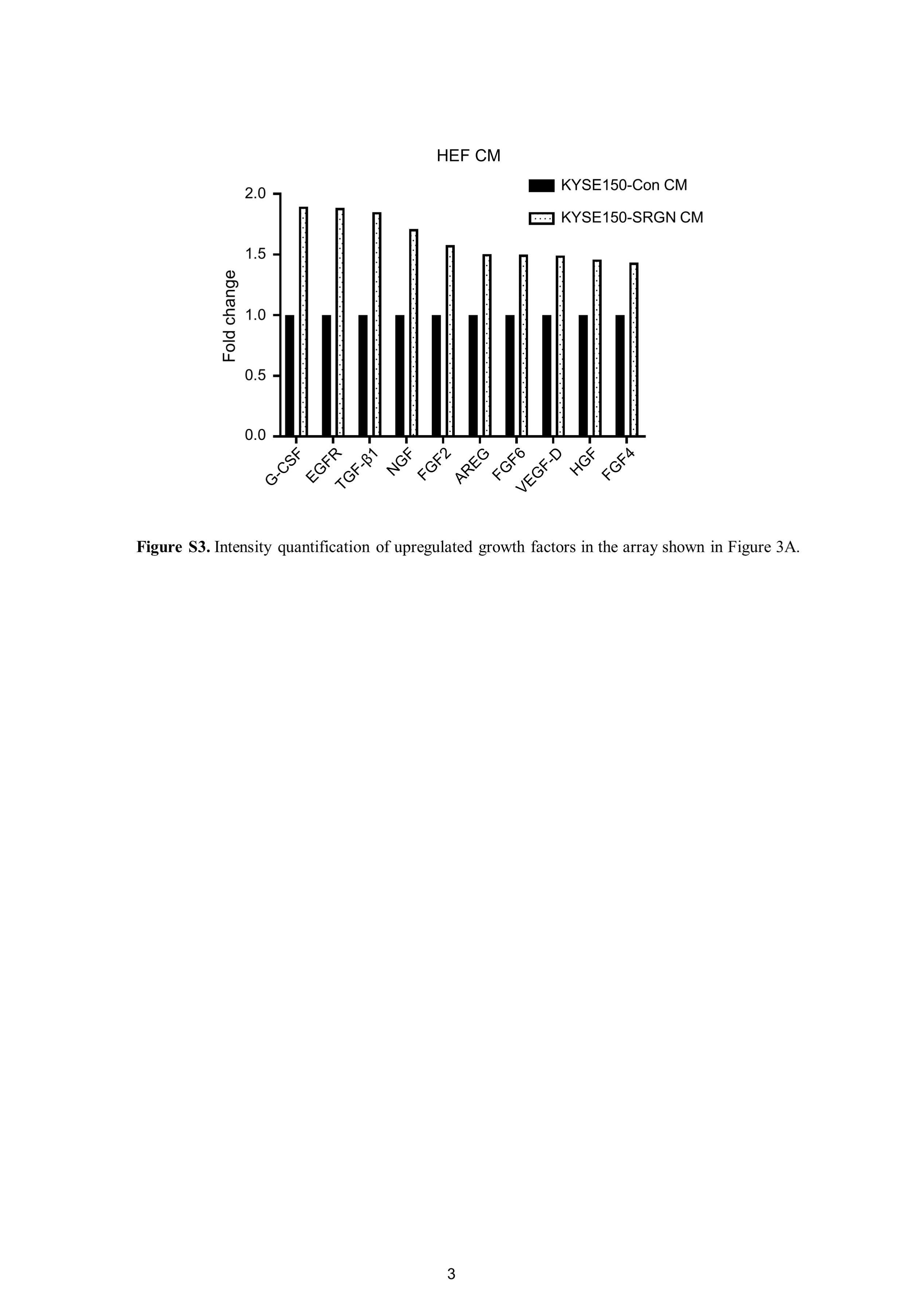

Supplement: Supplementary file 5 — Supporting Information [file CTM2-12-e1031-s009.jpg]

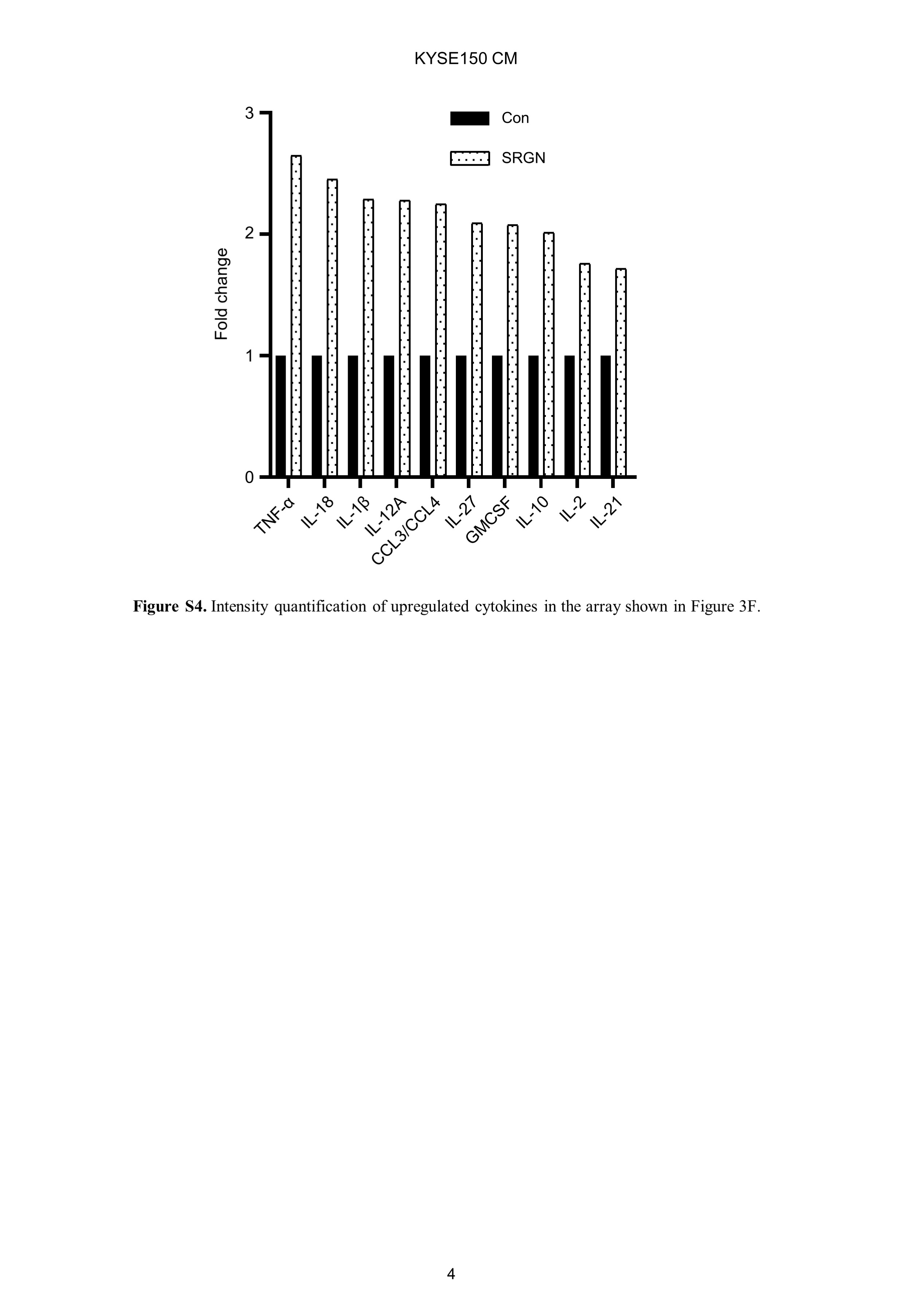

Supplement: Supplementary file 6 — Supporting Information [file CTM2-12-e1031-s007.jpg]

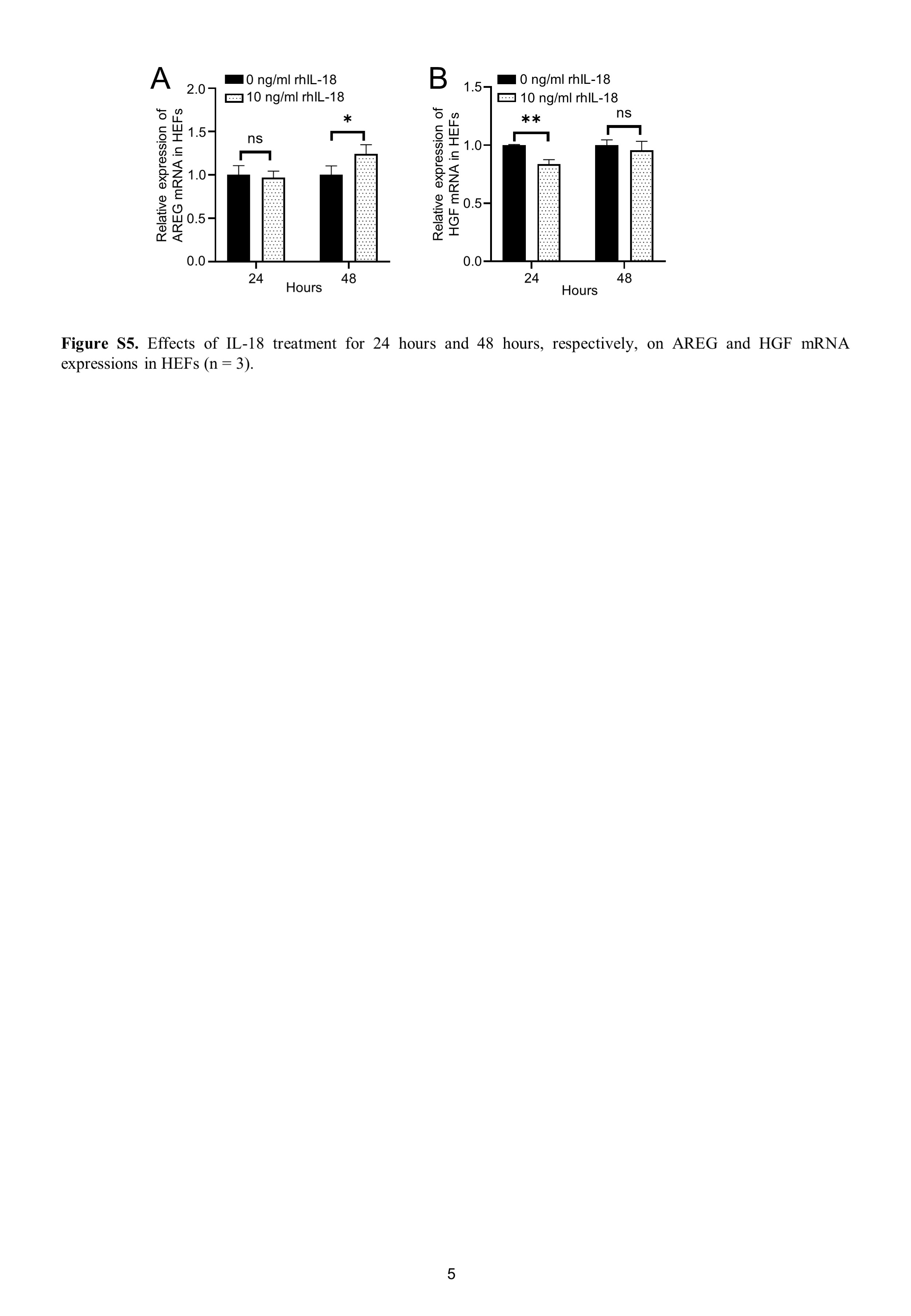

Supplement: Supplementary file 7 — Supporting Information [file CTM2-12-e1031-s010.jpg]

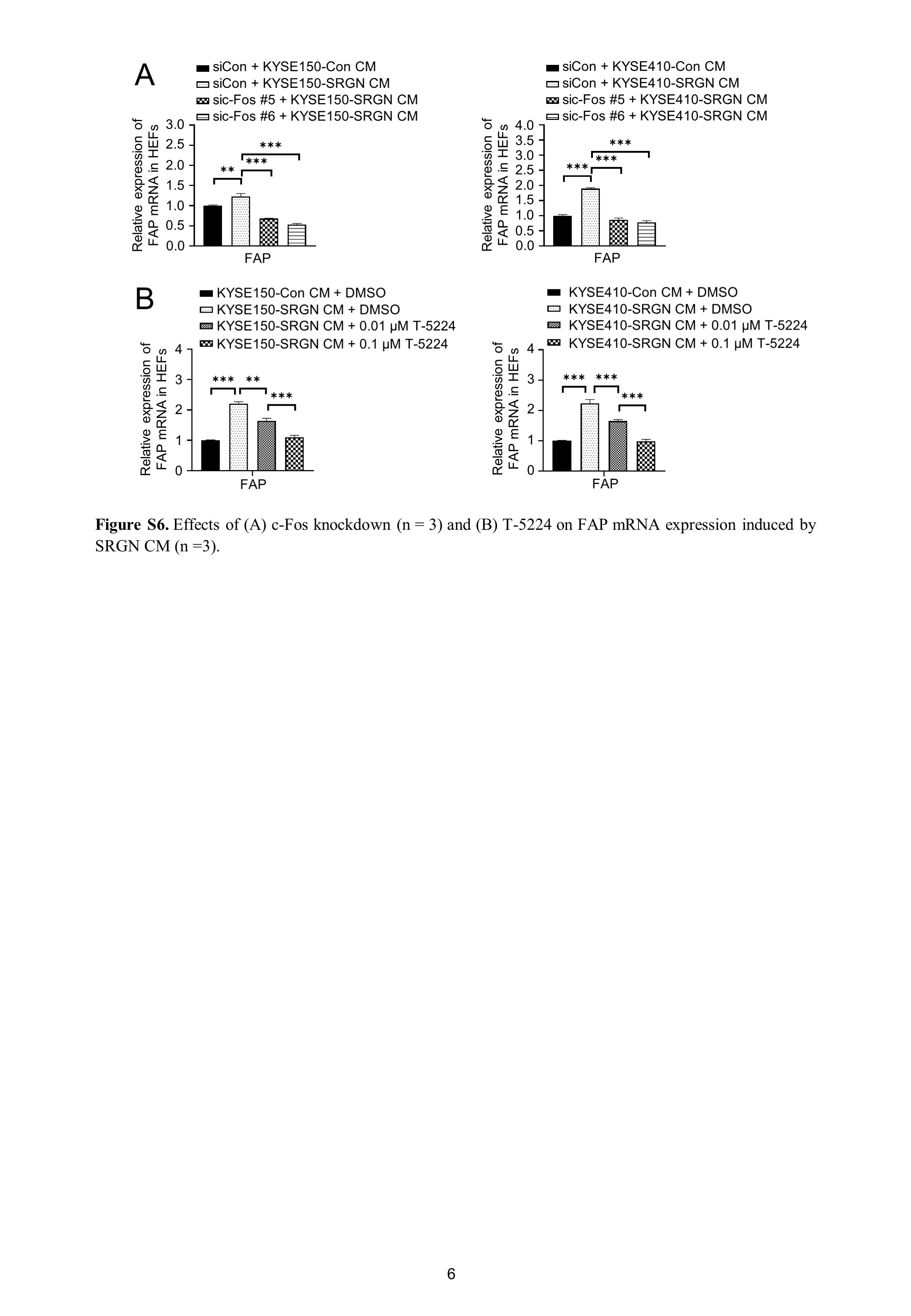

Supplement: Supplementary file 8 — Supporting Information [file CTM2-12-e1031-s008.jpg]

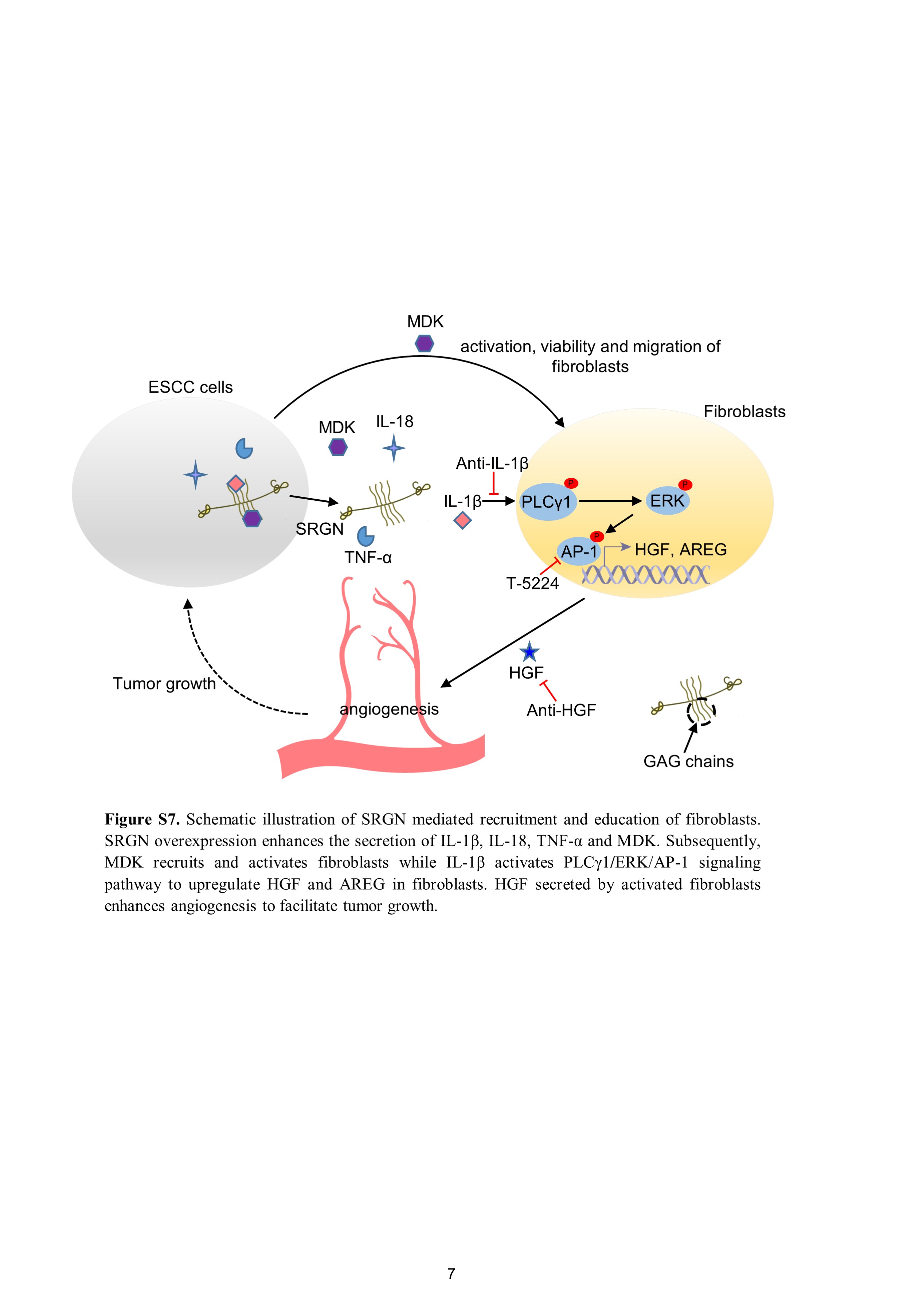

Supplement: Supplementary file 9 — Supporting Information [file CTM2-12-e1031-s006.jpg]

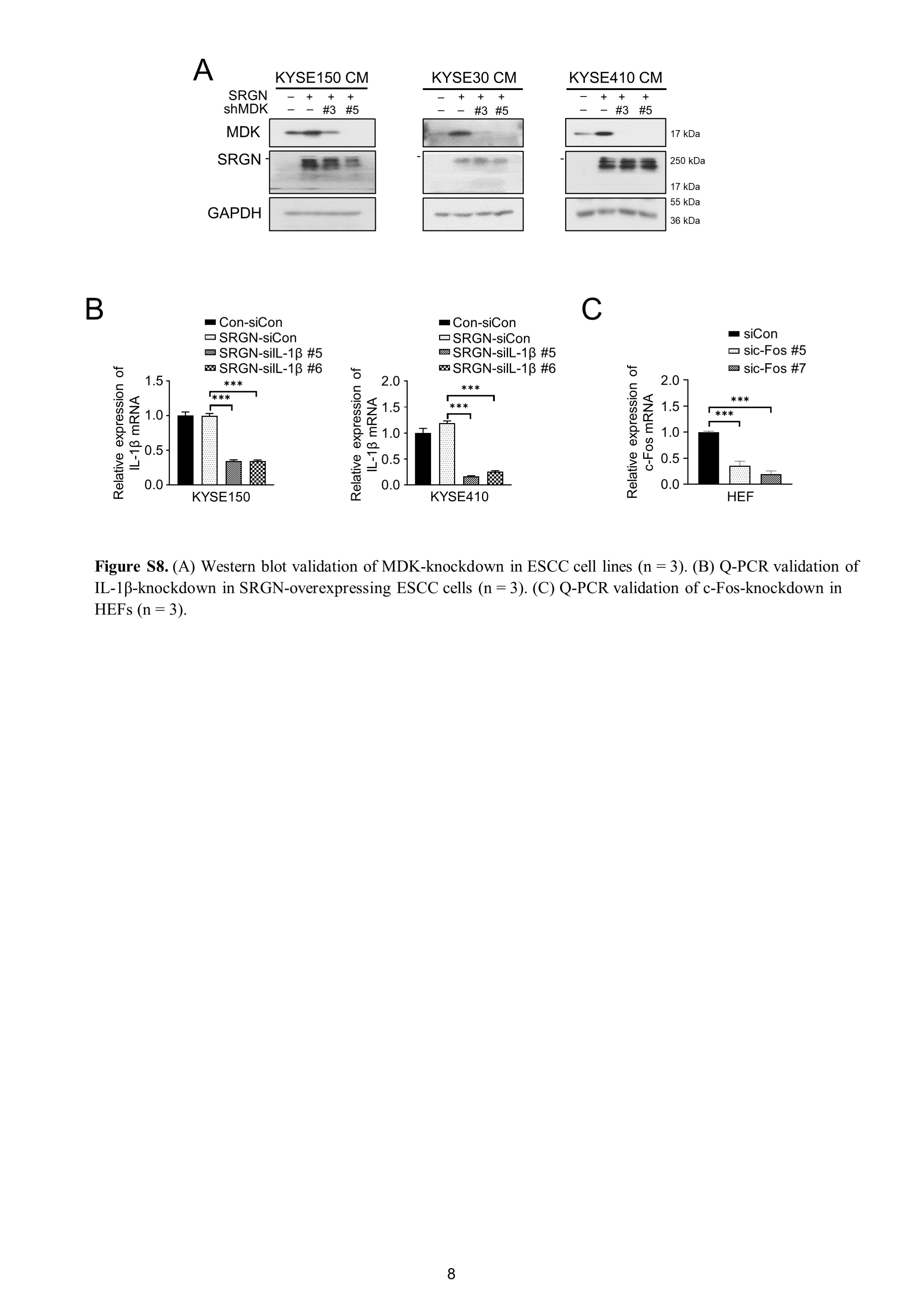

Supplement: Supplementary file 10 — Supporting Information [file CTM2-12-e1031-s005.jpg]
